# The association of sexual minority status and bullying victimization is modified by sex and grade: findings from a nationally representative sample

**DOI:** 10.1186/s12889-024-17988-y

**Published:** 2024-02-16

**Authors:** Junjie Lu, Jiarui Yang, Ekaterina Sadikova, Henning Tiemeier

**Affiliations:** 1 Department of Social and Behavior Sciences, Harvard University T.H. Chan School of Public Health, Boston, USA; 2 Department of Radiology, Massachusetts General Hospital, Harvard Medical School, Boston, USA; 3 Department of Epidemiology, Harvard University T.H. Chan School of Public Health, Boston, USA

**Keywords:** Bullying victimization, Sexual minority, Sex, Effect modification

## Abstract

**Background:**

Sexual minority status is associated with face-to-face bullying and cyberbullying victimization. However, limited studies have investigated whether such a relationship differs by sex or grade in a nationally representative sample.

**Methods:**

We concatenated the national high school data from the Youth Risk Behavior Surveillance System (YRBSS) chronologically from 2015 to 2019, resulting in a sample of 32,542 high school students. We constructed models with the interaction term between sexual minority status and biological sex assigned at birth to test the effect modification by sex on both the multiplicative and additive scales. A similar method was used to test the effect modification by grade.

**Results:**

Among heterosexual students, females had a higher odds of being bullied than males, while among sexual minority students, males had a higher odds of being bullied. The effect modification by sex was significant on both the multiplicative and additive scales. We also found a decreasing trend of bullying victimization as the grade increased among both heterosexual and sexual minority students. The effect modification by the grade was significant on both the multiplicative and the additive scale.

**Conclusions:**

Teachers and public health workers should consider the difference in sex and grade when designing prevention programs to help sexual minority students.

**Supplementary Information:**

The online version contains supplementary material available at 10.1186/s12889-024-17988-y.

## Background

### Prevalence and consequences of bullying

Bullying is characterized by a systematic abuse of power, involving aggressive behavior or intentional harm-doing by peers, carried out repeatedly, and encompassing an imbalance of power between the victim and the bully [1]. Approximately one in four American high school students experienced bullying in 2019 [2]. Cyberbullying, distinct from traditional bullying, is any form of bullying conducted via electronic means, such as mobile phones or the internet, and has gained increasing attention. Reports indicate that 10–14% of young individuals experience chronic bullying lasting more than six months [3, 4].

The public health concern of bullying arises due to its association with deteriorating mental and physical health, increased school phobia, poorer academic performance, and heightened suicide risk [5–7].

### Disparities in bullying victimization among sexual minorities

Sexual minority youth, including lesbian, gay, bisexual, transgender, and questioning (LGBTQ) individuals, experience higher rates of both traditional and cyberbullying victimization compared to their heterosexual counterparts, exacerbating the health inequality gap [8, 9]. Evidence suggests that LGBQ females and LGBQ males experience higher rates of bullying than heterosexual males [10]. Moreover, studies examining whether sexual minority youth are targeted due to their sexual orientation or gender identity have found high numbers of youth reporting or witnessing homophobic bullying. According to the 2013 National School Climate Survey, among LGBT students, 74.1% reported verbal harassment, 36.2% reported physical harassment, and 49.9% reported electronic harassment due to their perceived sexual orientation [11].

### Effect modification by sex

Significant sex differences have been identified in traditional bullying behaviors. Boys are more likely to be involved in bullying compared to girls [12]. Regarding the types of bullying, boys are more prone to engage in physical and verbal direct forms of bullying, while girls tend to participate in relationship bullying, such as spreading rumors and making sexual comments [13]. Sex differences in cyberbullying remain inconclusive. Some studies suggest that girls may engage more in cyberbullying than traditional forms due to their inclination towards relation-related violence. Specifically, girls may spread rumors, gossip, and exclude other girls online anonymously, to establish close friendships while promoting bullying behavior [14]. Conversely, other researchers have found that boys are more likely to engage in cyberbullying as well [15]. This discrepancy may arise because girls are more likely to report cyberbullying incidents to parents, teachers, or researchers, while boys may conceal their victimization [16].

Various theories have been proposed to explain gender differences in bullying. One emphasizes the development of masculinity among boys, driving them to seek power and leadership within peer groups, which in turn stimulates aggressive behaviors, including bullying [17, 18]. Another theory highlights gender stereotypes, positing that girls who exhibit aggressive behavior may be perceived as violating normative expectations and even as immature or norm-breaking [19].

### Effect modification by school grade

Age-related differences in bullying have been reported in several studies, with bullying behavior peaking during middle school (12–15 years) and decreasing by the end of high school [20]. As age increases, direct and physical forms of bullying are replaced by indirect and relational forms, resulting in more subtle and complex bullying manifestations [21]. The decline in bullying with age may be attributed to younger students being more vulnerable to victimization due to their weaker position compared to older students [22]. Meanwhile, cognitive theory suggests that younger students lack a clear understanding of the criteria constituting bullying, such as intent, repetition, and power imbalance. As they mature, students develop a more defined understanding of bullying behavior, leading to a decrease in bullying as they establish moral standards [23, 24].

### Current study

Although researchers have identified sex differences in bullying behavior, a nationally representative sample should be employed to officially examine the interaction between sex and sexual minority status in bullying victimization on both the multiplicative and additive scale. Formal statistical tests are also needed to explore the effect of school grade on the bullying victimization of sexual minority students.

Thus, the objectives of this study are to: (a) investigate the effect modification by sex on the association between sexual minority status and bullying victimization; and (b) examine the effect modification by school grade on the association between sexual minority status and bullying victimization. Through this study, we aim to: (a) inform targeted interventions for bullying prevention; (b) contribute to a better understanding of the diverse experiences of sexual minority youth; and (c) guide policy and practice in schools and communities.

## Methods

### Study sample and population

The Youth Risk Behavioral Surveillance Surveys (YRBSS) is a national survey dataset aiming to monitor priority health risk behaviors that contribute markedly to the leading causes of death, disability, and social problems among youth and adults in the United States [25]. We concatenated cross-sectional data from the 2015, 2017, and 2019 YRBSS data for nation-wide high school students from 9th to 12th grade. 44,066 students completed YRBSS questionnaire from 2015 to 2019. Among them, 11,524 individuals missed one or more variables used in this study. Sample characteristics were shown in Table 1.

**Table 1:** Descriptive Statistics of the High School Sample Stratified by Sex Assigned at Birth (YRBSS, 2015–2019)

	Overall (*n* = 32,542, 100%)	Females (*n* = 16,360, 50.3%)	Males (*n* = 16,182,49.7%)
**Experienced on-site or cyber bullying in the Past 12 Months (%)**			
Yes	24.5	19.4	29.7
**Sexual Minority Status** ^**a**^ **(%)**			
Yes	12.9	7.1	18.7
**Race/Ethnicity (%)**			
White	55.0	55.3	54.6
Hispanic/Latino	9.7	9.5	9.9
Black or African American	11.7	11.8	11.5
Other ^b^	23.7	23.4	24.0
**Grade (%)**			
9th Grade	26.6	27.5	25.6
10th Grade	25.4	24.9	25.8
11th Grade	24.0	23.9	24.2
12th Grade	23.9	23.6	24.3
Ungraded or other grade	0.082	0.093	0.070
**Feeling sad or hopeless** ^**c**^ **(%)**			
Yes	32.5	41.6	23.5
**Past 30-Day Tobacco Use** ^**d**^ **(%)**			
Yes	26.9	28.0	25.8
**Past 30-Day Alcohol Use (%)**			
Yes	30.4	29.0	31.7
**Past 30-Day Marijuana Use (%)**			
Yes	20.1	20.6	19.7
**Year (%)**			
2015	38.5	39.0	37.9
2017	29.7	29.4	30.2
2019	31.8	31.6	32.1

^a^ Students are sexual minorities if they self-identified as gay, lesbian, bisexual, or not sure

^b^ Other races/ethnicities include Asian, Native Hawaiian/Other Pacific Islanders, and other multiple races/ethnicities

^c^ Students were asked ever feel so sad or hopeless almost every day for two weeks or more in a row that you stopped doing some usual activities

^d^ Students who self-reported ever using one of the following products during the past 30 days: cigarettes, electronic cigarettes, cigar products

This was an observational study using publicly available secondary data, and all personal information was de-identified [25]. As a result, no additional institution review (IRB) approval was required.

### Measures

#### Bullying

The study’s outcome was ever being bullied on school property or virtually in the past 12 months. Students were asked: “During the past 12 months, have you ever been bullied on school property?” and “During the past 12 months, have you ever been virtually bullied? (Count being bullied through texting, Instagram, Facebook, or other social media.).” Those who answered “Yes” to at least one of these questions were considered to experience school or cyberbullying victimization during the past 12 months.

#### Sexual minority status

The primary independent variable was the sexual minority status. Students were asked: “Which of the following best describes you?” Possible options include “Heterosexual (straight),” “Gay or lesbian,” “Bisexual,” and “Not sure.” Those who self-identified as “Gay or lesbian,” “Bisexual,” or “Not sure” were categorized as “sexual minorities.”

#### Sex assigned at birth

We examined the biological sex assigned at birth as the effect modifier of the association between the sexual minority status and bullying victimization at school or virtually in the past 12 months. Students were asked, “What is your sex?” Answer options were “female” and “male”. We coded the sex as a binary variable.

#### Grade

School grade was examined as a possible effect modifier. Students were asked “In what grade are you?” The school grade was coded as a continuous variable ranging from 9th to 12th grade.

#### Covariates

We controlled for demographic covariates to reduce confounding. Race/ethnicity was coded as a categorical variable, including White (reference group), Hispanic/Latino, Black or African American, and other races/ethnicities (Asian, American Indian/Alaska Native, Native Hawaiian/Other Pacific Islanders, and multiple races/ethnicities).

The study also controlled for variables including mental health conditions and substance use in additional analyses as these variables may also be viewed as mediators. Students were asked whether they ever felt so sad or hopeless almost every day for two weeks or more in a row that they stopped doing some usual activities. Answers were dichotomized as “Yes” and “No”. For self-reported substance use, consuming at least one drink on one or more days during the past 30 days was considered as currently drinking alcohol. Additionally, students were considered as currently using marijuana if they self-reported 1 or more times used marijuana during the past 30 days. The use of tobacco during the past 30 days was also included in the model. Students who self-reported ever using cigarette, e-cigarettes, or cigars during the past 30 days were considered as current tobacco users.

We include these covariates because both mental health conditions and substance use behaviors had close associations with the sexual minority status and bullying behaviors [26–29]. We also performed the analysis without mental health indicator and substance use. The results were shown in the Supplementary Material.

### Statistical analysis

We constructed two models to test the possible effect modification between the sexual minority status and bullying victimization during the past 12 months by biological sex at birth and grade. In the first model, we generated a product term between biological sex assigned at birth and sexual minority status. To test effect modification by biological sex assigned at birth on the multiplicative scale, we performed a Wald’s test to examine whether the coefficient of the product term differed from 0. To test effect modification by biological sex assigned at birth on the additive scale, the relative excess risk due to interaction (RERI) was calculated [30]. In the second model, similar product terms between grade and the sexual minority status were generated and again effect modification on both the multiplicative and additive scale was tested. The significant level was set at 0.05 for two-sided tests.

To address missing values in our data, we employed the multiple imputation method, specifically using fully conditional specification (FCS) with predictive mean matching (pmm) implemented in Multiple Imputation by Chained Equations (MICE). This approach led to the generation of 20 imputed datasets for thorough analysis. A complete case analysis was done as a sensitivity analysis and its congruence to the primary analysis provides confidence in our findings. The findings derived from this complete case analysis are comprehensively presented in the Supplementary Material.

We used the “*svy*” command and weight, stratum, and primary sampling unit variables in Stata to account for the complex sampling design of YRBSS and made the sample nationally representative. All the analyses were performed using the Stata/SE Version 16 (College Station, TX).

## Results

### Descriptive statistics

Table 1 showed the descriptive statistics for the overall study sample and stratified by sex assigned at birth. Among the individuals in the sample, 24.5% (95% CI: 23.6–25.5) of high school students reported having experienced onsite or cyberbullying during the past 12 months. In this sample, 12.9% (95% CI: 12.0–13.8) of high school students self-identified as sexual minorities, 7.1% (95% CI: 6.5–7.8) of females and 18.7% (95% CI: 17.2–20.0) of males self-reported being a sexual minority. Further, 19.4% (95% CI: 18.4–20.4) of females and 29.7% (95% CI: 28.4–31.0) of males self-reported having experienced onsite or cyberbullying.

### Analytic statistics

#### Effect modification by biological sex assigned at birth

**Fig. 1:**
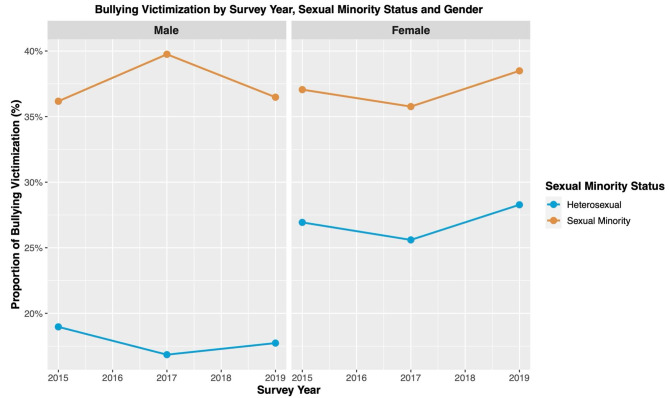
Bullying victimization by survey year, sexual minority status and biological sex assigned at birth (YRBSS, 2015–2019)

Table 2; Fig. 1 showed the effect modification of the relationship between sexual minority status and bullying victimization by biological sex assigned at birth. Among heterosexual adolescents, we found that 18.54% (95% CI: 17.62–19.45) of males and 27.98% (95% CI: 26.63–29.33) of females experienced bullying victimization, while among the sexual minority adolescents the numbers were 35.24% (95% CI: 31.86–38.63) for males and 38.71% (95% CI: 36.21–41.22) for females.

**Table 2:** Modification of the effect of sexual minority status on bullying victimization by biological sex assigned at birth

Total population analyses ^1^			
		Percentage of being bullied	Adjusted OR (95% CI) ^2^
**Heterosexual**	Male	18.54%	1.0, Reference
	Female	27.98%	1.46 (1.34–1.58), *p* < 0.001
**Sexual Minority**	Male	35.24%	1.93 (1.63–2.24), *p* < 0.001
	Female	38.71%	1.78 (1.56–2.00), *p* < 0.001
**Stratified analyses by sex at birth** ^**1**^			
	**Male**	Heterosexual	1.0, Reference
		Sexual Minority	1.92 (1.64–2.26), *p* < 0.001
	**Female**	Heterosexual	1.0, Reference
		Sexual Minority	1.22 (1.08–1.37), *p* = 0.001

Note:

1. *Total population analysis uses heterosexual males as the common reference group. Stratified analyses conducted separately for male and female students, both utilizing heterosexual students as their respective reference groups*

2. *ORs were adjusted for race, grade, feeling sad or hopeless, and the use of the following products during the past 30 days: cigarettes, electronic cigarettes, and cigars*

3. *Measure of effect modification on the additive scale (RERI): -0.62 (-0.96 - -0.28), p = 0.001*

4. *Measure of effect modification on the multiplicative scale: 0.63 (0.52–0.76), p < 0.001*

**Fig. 2:**
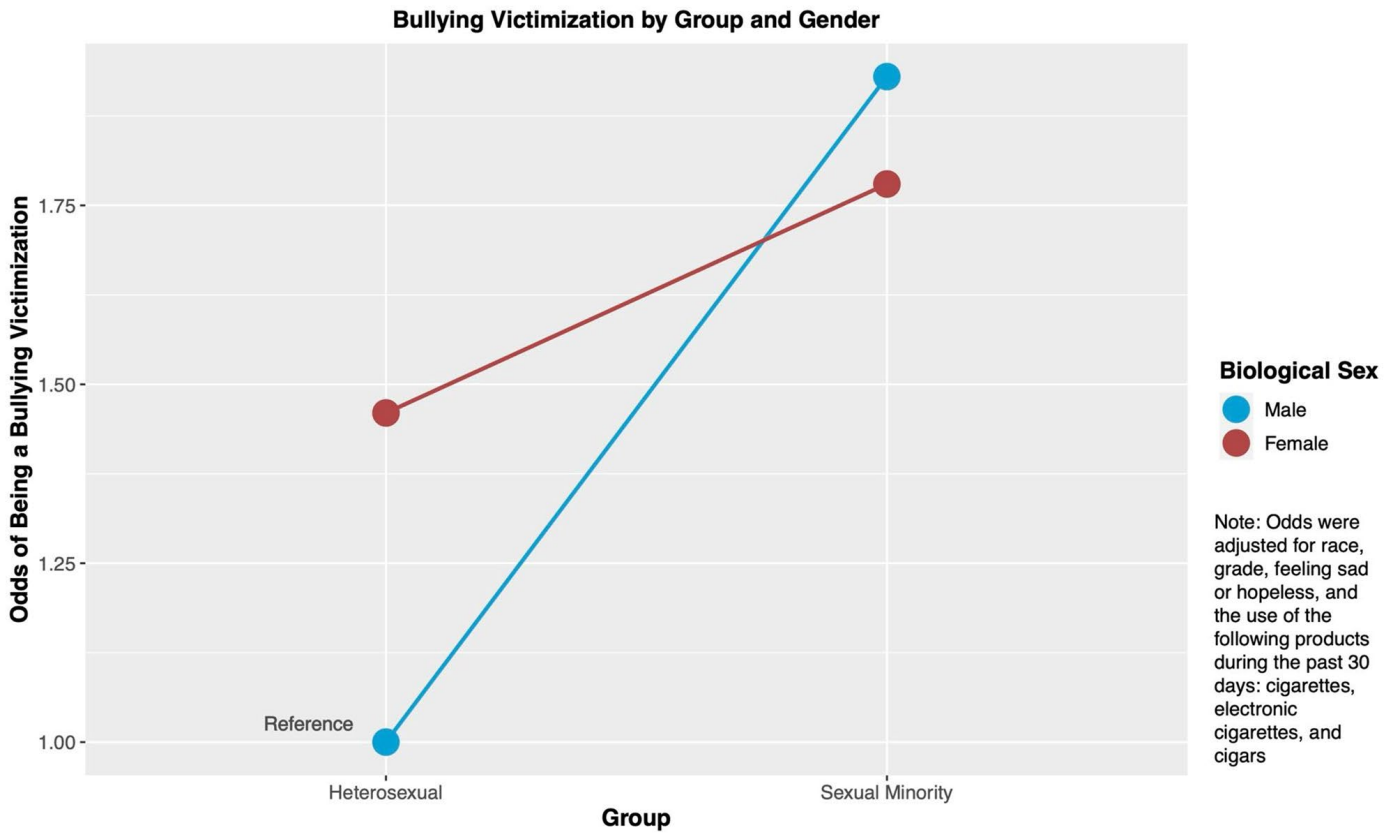
Odds of Bullying victimization by sexual minority status and biological sex (YRBSS, 2015–2019)

Using the male heterosexual adolescents as the reference group, we found that male sexual minority adolescents had the highest OR of being bullied (OR = 1.93; 95% CI: 1.63–2.24; *p* < 0.001) compared with female heterosexuals (OR = 1.46; 95% CI: 1.34–1.58; *p* < 0.001) and female sexual minorities (OR = 1.78; 95% CI: 1.56–2.00; *p* < 0.001). Among heterosexuals, females had higher odds of being bullied than males after adjusting for race, grade, feeling sad or hopeless, and the substance usage during the past 30 days. However, among sexual minorities, males had a higher odd of being bullied compared with females and adjusted for the same variables, as shown in Fig. 2.

When we stratified the sample by the biological sex assigned at birth, we found that among males, sexual minority adolescents had 1.92 times the odds (95% CI: 1.64–2.26; *p* < 0.001) of being the victim of bullying compared with heterosexuals. In a similar analysis among females, the OR was 1.22 (95% CI: 1.08–1.37; *p* = 0.001).

Next, we formally examined the effect modification by biological sex assigned at birth on the association between sexual minorities and bullying victimization, we found that on the multiplicative scale, the ratio of ORs was 0.63 (95% CI: 0.52–0.76; *p* < 0.001). This meant that sexual minority status increased the likelihood of bullying victimization among females less than among males. On the additive scale, RERI was– 0.62 (95% CI: -0.96 - -0.28, *p* = 0.001). Since this value was smaller than 0, there was negative effect modification of the sexual minority status across strata of the biological sex assigned at birth on an additive scale. In other words, this showed that the estimated impact of sexual minority status among females was smaller than that among males.

#### Effect modification by grade

**Fig. 3:**
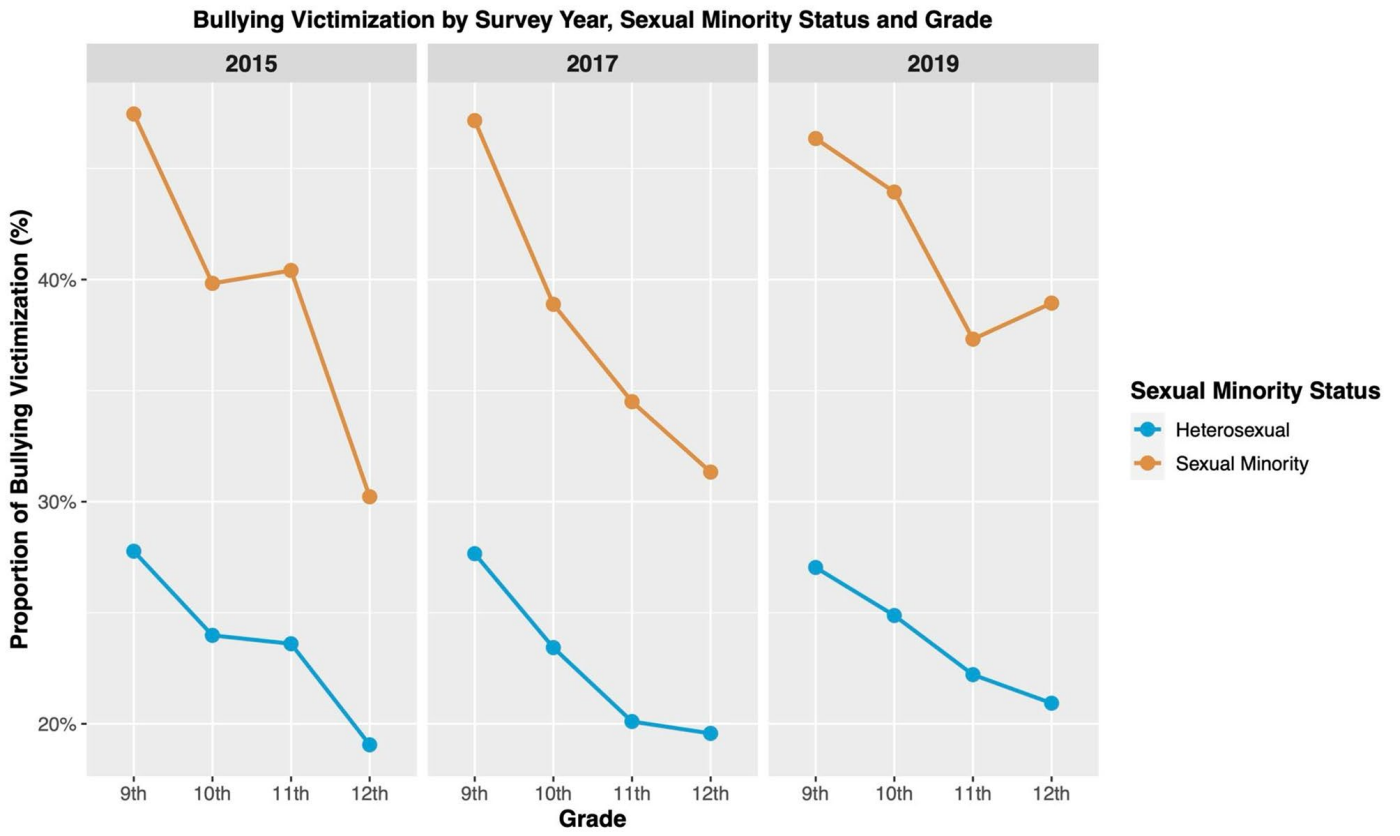
Bullying victimization by survey year, sexual minority status and grade (YRBSS, 2015–2019)

Table 3; Fig. 3 showed the association of sexual minority status with bullying victimization stratified by grade. There was a trend of decrease in experiencing bullying victimization with higher grades, and this trend was observed among both the heterosexual and sexual minority adolescents.

**Table 3:** Modification of the effect of sexual minority status on bullying victimization by grade

Total population analyses ^1^			
		Percentage of being bullied	Adjusted OR (95% CI) ^2^
**Heterosexual**	12th Grade	19.9%	1.0, Reference
	11th Grade	21.3%	1.43 (1.33–1.55), *p* < 0.001
	10th Grade	24.2%	1.73 (1.52–1.93), *p* < 0.001
	9th Grade	25.7%	2.07 (1.75–2.39), *p* < 0.001
**Sexual Minority**	12th Grade	31.5%	1.32 (1.06–1.58), *p* = 0.007
	11th Grade	36.5%	1.99 (1.67–2.30), *p* < 0.001
	10th Grade	38.5%	2.43 (2.07–2.80), *p* < 0.001
	9th Grade	42.4%	2.98 (2.44–3.53), *p* < 0.001
**Stratified analyses by school grades** ^**1**^			
	**12th Grade**	Heterosexual	1.0, Reference
		Sexual Minority	1.27 (1.03–1.56), *p* = 0.023
	**11th Grade**	Heterosexual	1.0, Reference
		Sexual Minority	1.47 (1.20–1.79), *p* < 0.001
	**10th Grade**	Heterosexual	1.0, Reference
		Sexual Minority	1.34 (1.09–1.66), *p* = 0.007
	**9th Grade**	Heterosexual	1.0, Reference
		Sexual Minority	1.48 (1.26–1.74), *p* < 0.001

Note:

1. *Total population analysis uses heterosexual students in 12th Grade as the common reference group. Stratified analyses conducted separately for students in 12th, 11th, 10th, and 9th Grade, both utilizing heterosexual students as their respective reference groups*

2. *ORs were adjusted for race, sex, feeling sad or hopeless, and the use of the following products during the past 30 days: cigarettes, electronic cigarettes, and cigars*

3. *Measure of effect modification on the additive scale (RERI) and using students in the 12th Grade as the reference group: 11th Grade: 0.22 (0.03–0.42), p = 0.021; 10th Grade: 0.39 (0.02–0.76), p = 0.041; 9th Grade: 0.59 (-0.04–1.22), p = 0.067*

4. *Measure of effect modification on the multiplicative scale and using students in the 12th Grade as the reference group: 11th Grade: 1.04 (0.86–1.22), p < 0.001; 10th Grade: 1.07 (0.79–1.35), p < 0.001; 9th Grade: 1.09 (0.71–1.48), p < 0.001*

**Fig. 4:**
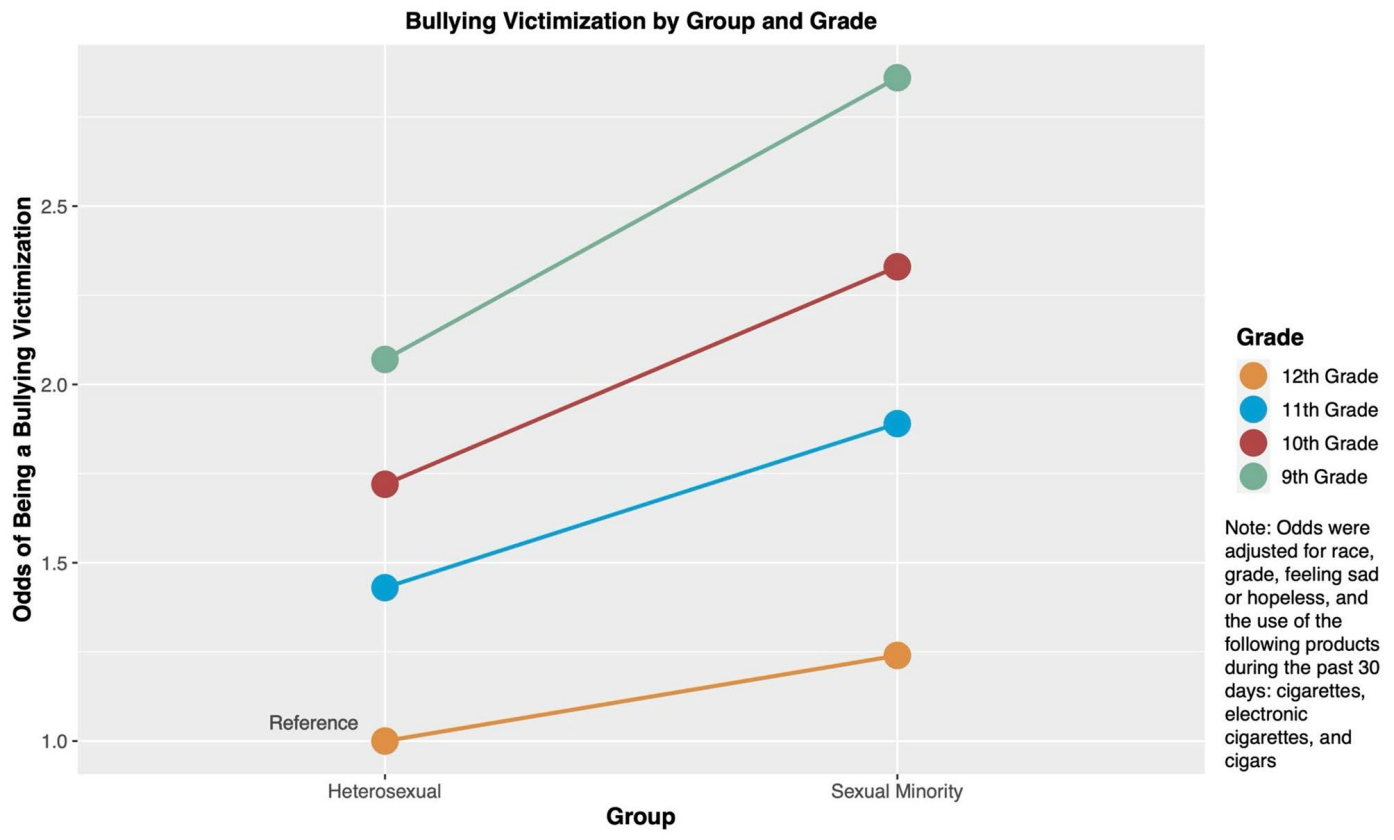
Odds of Bullying victimization by sexual minority status and grade (YRBSS, 2015–2019)

Using the 12th grade heterosexual adolescents as the reference group, which had the lowest odds of being the bullying victimization, we found that 9th grade sexual minority adolescents had the highest odds of being the bullying victimization (OR: 2.98; 95% CI: 2.44–3.53; *p* < 0.001), adjusting for race, grade, feeling sad or hopeless, and the use of tobacco products during the past 30 days. Additionally, we discovered that the relationship between sexual minority status and being bullying victimization was influenced by the grade level of students. When using 12th Grade as the reference group, there was a decrease in the likelihood of bullying victimization in 11th, 10th, and 9th Grades. The odds ratios for these grades were 1.04 (0.86–1.22, *p* < 0.001) for 11th Grade, 1.07 (0.79–1.35, *p* < 0.001) for 10th Grade, and 1.09 (0.71–1.48, *p* < 0.001) for 9th Grade. Furthermore, we observed a significant effect of grade level on the additive scale for both 11th and 10th Grades, but not for 9th Grade, when compared to the 12th Grade. The RERI values were 0.22 (0.03–0.42, *p* = 0.021) for 11th Grade, 0.39 (0.02–0.76, *p* = 0.041) for 10th Grade, and 0.59 (-0.04–1.22, *p* = 0.067) for 9th Grade. These findings were shown in Fig. 4.

## Discussion

This study discovered that the relationship between sexual minority status and experiencing onsite or cyberbullying in the past 12 months was significantly influenced by biological sex assigned at birth in a nationally representative sample. Among heterosexual individuals, females exhibited a higher likelihood of experiencing bullying compared to males. However, among sexual minorities, males faced a higher likelihood of being bullied than females. This effect modification by biological sex assigned at birth was significant on both multiplicative and additive scales. Additionally, we observed a negative association between bullying victimization and school grade for both heterosexual and sexual minority adolescents, with a formal test of effect modification by grade proving significant.

Beginning in 2015, the YRBSS introduced questions about students’ sexual orientation in the national high school questionnaire. This study incorporated all available nationally representative data and was the first to explore the association between sexual minority status and onsite or cyberbullying victimization in this sample.

We found that 7.1% (95% CI: 6.5–7.8) of females and 18.7% (95% CI: 17.2–20.0) of males self-reported being a sexual minority. Several factors could elucidate our results. Culturally and socially, males who express non-heteronormative orientations or identities often face heightened stigma, which may paradoxically enhance their self-recognition as part of a minority and prompt more frequent self-reporting [31, 32]. Additionally, the distinct socialization patterns for male and female students—where males may perceive their divergence from gender norms as particularly pronounced—could intensify their identification with the sexual and gender minority (SGM) community [33]. Furthermore, the more prominent representation of male SGM figures in the media and public narratives may also play a role, fostering self-identification and the propensity to disclose such an identity [34, 35].

An increased likelihood of bullying victimization among heterosexual females compared to heterosexual males was also observed in a study involving 1,402 Brazilian students [36]. It is possible that female adolescents were more likely to express femininity in Brazilian cultural norms and thus appeared more physically fragile than males. Previous research also indicated that most bullying among girls occurred within friendship or acquaintanceship groups, suggesting that heightened bullying victimization among female heterosexuals may stem from emotions triggered by their friendships, such as jealousy, suspicion, disappointment, and anger [37]. Qualitative studies revealed that girls consider their friendships extremely important and nominated the breaking of a friendship as the most anxiety-provoking aspect of school life. Disputes and conflicts related to friendship among girls can be recognized as bullying incidents with clearly identified victims [37].

Our study was in line with previous studies. Researchers found that when asked about why they thought they were bullied in the past 3 months, male sexual minority students were more likely to list their sexual orientation or what others think about their sexual orientation and how they expressed their gender as reasons, compared to female sexual minority students [38]. Similar results were reported in a Rhode Island sample where sexual minority boys reported the higher odds of being recently bullied than heterosexual boys [39].

Several factors could explain the difference in bullying victimization odds between heterosexual and sexual minority students, including homophobia and heterosexism, gender role non-conformity, social acceptability of anti-gay pejoratives, and invisibility of sexual minorities [40–43]. Specifically, with heterosexuality dominating most schools, the gender expressions of sexual minority students, including those identifying as non-binary, deviated from their sex assigned at birth, triggering homophobia and anti-gay sentiments among students. It is also possible that sexual minority students assigned male at birth had limited participation in group activities. As group participation is a symbol of peer community belonging among high school students, limited engagement in group activities may cause male sexual minority students to appear distant from heterosexual peers, increasing the likelihood of being bullied [44].

Furthermore, the discrepancy in bullying victimization between male and female sexual minority students may be explained from a feminist perspective: male sexual minority students were often stereotyped as effeminate, and under the possible influence of misogyny, they were more vulnerable to becoming victims of bullying behavior [45]. Future studies could further evaluate the effects of these factors, as our study did not explore their possible roles in the observed outcomes.

In interpreting our findings, it was important to consider the baseline rates of bullying victimization. While our results indicate that sexual minority males have higher odds of being bullied compared to their heterosexual counterparts, this does not necessarily imply a higher absolute risk of bullying compared to sexual minority females. In fact, despite the lower odds ratio observed among sexual minority females compared to heterosexual females, the actual prevalence of bullying may be similar for sexual minority females and males. This was due to the higher baseline rate of bullying among males in general. Therefore, while sexual minority status increases the relative risk of bullying for both males and females, the absolute risk of bullying for sexual minority females and males might be more alike than the odds ratios alone suggest. This underscored the importance of considering both relative and absolute measures of risk in understanding the impact of sexual minority status on bullying victimization. Future research should further investigate these nuances to provide a more comprehensive understanding of bullying dynamics among adolescents.

We also found that although sexual minority students were more likely to be bullied, the situation improved as school grade increased. From a developmental psychology perspective, older individuals may be more psychologically mature than younger people and more likely to accept social norms, including showing greater respect for others, being less aggressive, and paying more attention to interpersonal relationships, which may contribute to a general decrease in bullying [46]. However, this finding was derived from an imputation method and when we analyzed the full data set, the interaction on the multiplicative scale disappeared. This indicated that the observed trend may not be exclusive to sexual minority students, but rather in line with a general trend in the school environment.

Our findings emphasize the importance of considering biological sex assigned at birth when developing strategies to prevent bullying among sexual minority adolescents. In a previous review, 44 interventions aimed at reducing stress among sexual minorities were systematically examined. Although all of these interventions were designed to promote bullying behavior change at multiple levels, few considered biological sex assigned at birth as a factor when designing the intervention [47]. Our study suggests that considering heterogeneity and underlying mechanisms between male and female students could potentially increase the efficacy and effectiveness of bullying behavior interventions.

This study has some limitations. First, other variables associated with both sexual minority status and bullying victimization, such as childhood adverse experiences [26, 48, 49], were not available in the dataset. Comparable constraints were present in the sex variable, as YRBS inquired about “sex” rather than explicitly focusing on “sex assigned at birth.” Consequently, we are unable to pinpoint young individuals who do not conform to the binary classification. Future research should utilize a more all-encompassing variable to encompass this data.

Besides, students selecting ‘Not sure’ in the sexual orientation question may have been uncertain regarding the wording of the question rather than their sexual orientation. YRBSS lacks an alternative for expressing ambiguity about the question’s phrasing, such as ‘I am unsure about the question.’ Consequently, we presumed that students opting for ‘Not sure’ were ambiguous about their sexual orientation. This assumption could result in misclassification of the ‘exposure’ variable, in this case, sexual orientation, potentially leading to a bias in our results towards the null hypothesis.

In addition, in our effort to streamline analyses and align with public health intervention strategies that typically address broader groups, we categorized “Gay or Lesbian” and “Bisexual” identities into a single “sexual minority” variable. While this facilitated a clear comparative analysis, we recognize it may not fully capture the distinct experiences within each subgroup. Consequently, our study further implemented stratification analysis across different subgroups within sexual minorities. This analysis revealed a significant sex-based effect modification on the multiplicative scale for both gay/lesbian and bisexual students. In contrast, on the additive scale, this sex-based effect modification was significant only in the comparison between gay/lesbian and heterosexual students, while it was not significant when comparing bisexual and heterosexual students. Furthermore, when assessing the effect modification by grade level, our findings indicated a significant grade-based effect modification on the multiplicative scale for both gay/lesbian and bisexual students. However, this grade-based effect modification was not significant on the additive scale when comparing gay/lesbian and bisexual students to their heterosexual counterparts. Comprehensive details of these findings are provided in the supplementary material.

We also recognize that treating sex and sexual identity as independent variables may oversimplify the complex interplay between these identities. Intersectionality theory stipulates that these aspects of identity are interconnected and cannot be fully understood in isolation [7, 8]. Aligned with recommendations in intersectionality literature, we interpret the interaction between sex and sexual identity on the additive scale to assess whether intersecting elements of identity are greater than their sum and thus not independent [9].

Second, due to the self-reporting nature of the YRBSS questionnaire, this study was subject to recall bias. Furthermore, we only considered the victimization aspect of bullying behavior, even though bullying perpetration is also crucial in understanding and preventing bullying behavior [50]. However, acquiring such information is difficult using self-reported questionnaire data, as few students would admit to bullying others.

Moreover, our study primarily investigated sex and grade as effect modifiers to guide public health programs for students based on their sex assigned at birth and educational level. While race/ethnicity might also serve as an effect modifier, our focus was not on developing interventions for specific racial/ethnic groups, given the associated implementation complexities. Additionally, the YRBSS data used in our study does not comprehensively capture the nuances of race/ethnicity, which include both phenotypic traits and cultural identities, limiting our ability to fully explore its impact as a modifier.

It is important to acknowledge that the missingness in the sexual identity variable may not be random. This non-random nature of missing data is likely due to the reluctance or policy of many schools to include questions about sexual identity in their surveys, resulting in a systematic absence of this data. For sensitivity analysis, we conducted a complete case analysis. This additional analysis supports the robustness of our main conclusions, despite the noted potential bias in data collection.

## Conclusion

This study provided insight in the effect modification of the relationship between sexual minority status and face-to-face or cyber bullying victimization by biological sex assigned at birth and the school grade in a large nationally representative sample. Among heterosexual adolescents, females had higher odds of being bullied, however among sexual minority students, male adolescents had higher odds of being bullied. We witnessed decreasing bullying victimization as school grade increased in both the heterosexual and sexual minority students, and there was effect modification by grade on both multiplicative and additive scale. Public health workers and teachers should consider the factor of biological sex assigned at birth and the school grade when designing bullying behavior intervention plans targeting sexual minority students.

### Electronic supplementary material

Below is the link to the electronic supplementary material.

Supplementary Material 1

## Data Availability

The datasets generated and/or analyzed during the current study are available in the YRBSS repository, https://www.cdc.gov/healthyyouth/data/yrbs/index.htm.
