# Supplementary material for: The association of sexual minority status and bullying victimization is modified by sex and grade: findings from a nationally representative sample
Source: BMC Public Health. 2024 Feb 16;24:504. doi: 10.1186/s12889-024-17988-y (PMC10874033; doi:10.1186/s12889-024-17988-y)
Supplement: Supplementary file 1 — Supplementary Material 1 [file 12889_2024_17988_MOESM1_ESM.docx]

**Supplementary material**

**Model 1: Including the mental health variable and substance usage**

**Complete data analysis**

**Modification of the effect of sexual minority status on bullying victimization by biological sex assigned at birth**

| **Total population analyses ^1^** | | | |
| --- | --- | --- | --- |
|  |  | **Percentage of being bullied** | **Adjusted OR (95% CI) ^2^** |
| **Heterosexual** | Male | 18.54% | 1.0, Reference |
|  | Female | 28.21% | 1.51 (1.38 – 1.66), *p* < 0.001 |
| **Sexual Minority** | Male | 35.15% | 1.85 (1.50 - 2.30), *p* < 0.001 |
|  | Female | 38.09% | 1.77 (1.56 – 2.01), *p* < 0.001 |
| **Stratified analyses by sex at birth ^1^** | | | |
|  | **Male** | Heterosexual | 1.0, Reference |
|  |  | Sexual Minority | 1.85 (1.49 - 2.29), *p* < 0.001 |
|  | **Female** | Heterosexual | 1.0, Reference |
|  |  | Sexual Minority | 1.17 (1.03 – 1.33), *p* < 0.05 |

***Note:***

1. *Total population analysis uses heterosexual males as the common reference group. Stratified analyses conducted separately for male and female students, both utilizing heterosexual students as their respective reference groups.*
2. *ORs were adjusted for race, grade, feeling sad or hopeless, and the use of the following products during the past 30 days: cigarettes, electronic cigarettes, and cigars*
3. *Measure of effect modification on the additive scale (RERI): -0.60 (-1.05 - -0.15), p < 0.01*
4. *Measure of effect modification on the multiplicative scale: 0.63 (0.49 - 0.81), p < 0.001*

**Model 1: Including the mental health variable and substance usage**

**Complete data analysis**

**Effect of sexual minority status on bullying victimization by grade**

| **Total population analyses ^1^** | | | |
| --- | --- | --- | --- |
|  |  | **Percentage of being bullied** | **Adjusted OR (95% CI) ^2^** |
| **Heterosexual** | 12^th^ Grade | 19.8% | 1.0, Reference |
|  | 11^th^ Grade | 21.3% | 1.43 (1.32 – 1.59), *p* < 0.001 |
|  | 10^th^ Grade | 24.2% | 1.72 (1.51 – 1.96), *p* < 0.001 |
|  | 9^th^ Grade | 25.7% | 2.07 (1.74 – 2.46), *p* < 0.001 |
| **Sexual Minority** | 12^th^ Grade | 30.9% | 1.24 (0.94 – 1.66), *p* = 0.14 |
|  | 11^th^ Grade | 36.5% | 1.89 (1.58 – 2.26), *p* < 0.001 |
|  | 10^th^ Grade | 38.4% | 2.33 (1.96 – 2.77), *p* < 0.001 |
|  | 9^th^ Grade | 41.8% | 2.86 (2.33 – 3.52), *p* < 0.001 |
| **Stratified analyses by school grades ^1^** | | | |
|  | **12^th^ Grade** | Heterosexual | 1.0, Reference |
|  |  | Sexual Minority | 1.19 (0.95 – 1.50), *p* = 0.135 |
|  | **11^th^ Grade** | Heterosexual | 1.0, Reference |
|  |  | Sexual Minority | 1.36 (1.09 – 1.70), *p* < 0.01 |
|  | **10^th^ Grade** | Heterosexual | 1.0, Reference |
|  |  | Sexual Minority | 1.32 (1.04 – 1.67), *p* < 0.05 |
|  | **9^th^ Grade** | Heterosexual | 1.0, Reference |
|  |  | Sexual Minority | 1.42 (1.18 – 1.72), *p* < 0.001 |

***Note:***

1. *Total population analysis uses heterosexual students in 12^th^ Grade as the common reference group. Stratified analyses conducted separately for students in 12^th^, 11^th^, 10^th^, and 9^th^ Grade, both utilizing heterosexual students as their respective reference groups.*
2. *ORs were adjusted for race, sex, feeling sad or hopeless, and the use of the following products during the past 30 days: cigarettes, electronic cigarettes, and cigars*
3. *Measure of effect modification on the additive scale (RERI) and using students in the 12^th^ Grade as the reference group: 11^th^ Grade: 0.21 (0.01 – 0.40), p < 0.05; 10^th^ Grade: 0.36 (-0.03 – 0.74), p > 0.05; 9^th^ Grade: 0.55 (-0.11 – 1.21), p > 0.05.*
4. *Measure of effect modification on the multiplicative scale and using students in the 12^th^ Grade as the reference group: 11^th^ Grade: 1.05 (0.87 – 1.28), p > 0.05; 10^th^ Grade: 1.08 (0.81 – 1.45), p > 0.05; 9^th^ Grade: 1.11 (0.75 – 1.64), p > 0.05.*

**Model 2: Does not include the mental health variable and substance usage**

**Imputation analysis**

**Modification of the effect of sexual minority status on bullying victimization by biological sex assigned at birth**

| **Total population analyses ^1^** | | | |
| --- | --- | --- | --- |
|  |  | **Percentage of being bullied** | **Adjusted OR (95% CI) ^2^** |
| **Heterosexual** | Male | 18.54% | 1.0, Reference |
|  | Female | 28.21% | 1.71 (1.58 - 1.84), *p* < 0.001 |
| **Sexual Minority** | Male | 35.15% | 2.43 (2.04 - 2.82), *p* < 0.001 |
|  | Female | 38.09% | 2.85 (2.52 - 3.18), *p* < 0.001 |
| **Stratified analyses by sex at birth ^1^** | | | |
|  | **Male** | Heterosexual | 1.0, Reference |
|  |  | Sexual Minority | 2.43 (2.06 - 2.85), *p* < 0.001 |
|  | **Female** | Heterosexual | 1.0, Reference |
|  |  | Sexual Minority | 1.67 (1.50 - 1.87), *p* < 0.001 |

***Note:***

1. *Total population analysis uses heterosexual males as the common reference group. Stratified analyses conducted separately for male and female students, both utilizing heterosexual students as their respective reference groups.*
2. *ORs were adjusted for race and grade.*
3. *Measure of effect modification on the additive scale (RERI): -0.29 (-0.74 - 0.15), p > 0.05*
4. *Measure of effect modification on the multiplicative scale: 0.68 (0.57 – 0.82), p < 0.001*

**Model 2: Does not include the mental health variable and substance usage**

**Imputation analysis**

**Effect of sexual minority status on bullying victimization by grade**

| **Total population analyses ^1^** | | | |
| --- | --- | --- | --- |
|  |  | **Percentage of being bullied** | **Adjusted OR (95% CI) ^2^** |
| **Heterosexual** | 12^th^ Grade | 19.9% | 1.0, Reference |
|  | 11^th^ Grade | 21.3% | 1.30 (1.21 - 1.40), *p* < 0.001 |
|  | 10^th^ Grade | 24.2% | 1.49 (1.32 - 1.64), *p* < 0.001 |
|  | 9^th^ Grade | 25.7% | 1.69 (1.45 - 1.94), *p* < 0.001 |
| **Sexual Minority** | 12^th^ Grade | 31.5% | 1.71 (1.26 - 2.15), *p* < 0.001 |
|  | 11^th^ Grade | 36.5% | 2.38 (2.01 - 2.74), *p* < 0.001 |
|  | 10^th^ Grade | 38.5% | 2.81 (2.40 – 3.21), *p* < 0.001 |
|  | 9^th^ Grade | 42.4% | 3.31 (2.73 - 3.89), *p* < 0.001 |
| **Stratified analyses by school grades ^1^** | | | |
|  | **12^th^ Grade** | Heterosexual | 1.0, Reference |
|  |  | Sexual Minority | 1.74 (1.42 – 2.14), *p* < 0.001 |
|  | **11^th^ Grade** | Heterosexual | 1.0, Reference |
|  |  | Sexual Minority | 1.88 (1.55 – 2.29), *p* < 0.001 |
|  | **10^th^ Grade** | Heterosexual | 1.0, Reference |
|  |  | Sexual Minority | 1.79 (1.47 – 2.20), *p* < 0.001 |
|  | **9^th^ Grade** | Heterosexual | 1.0, Reference |
|  |  | Sexual Minority | 1.98 (1.68 – 2.32), *p* < 0.001 |

***Note:***

1. *Total population analysis uses heterosexual students in 12^th^ Grade as the common reference group. Stratified analyses conducted separately for students in 12^th^, 11^th^, 10^th^, and 9^th^ Grade, both utilizing heterosexual students as their respective reference groups.*
2. *ORs were adjusted for race and sex.*
3. *Measure of effect modification on the additive scale (RERI) and using students in the 12^th^ Grade as the reference group: 11^th^ Grade: 0.37 (0.12 – 0.61), p < 0.01; 10^th^ Grade: 0.61 (0.16 – 1.07), p < 0.01; 9^th^ Grade: 0.91 (0.17 – 1.64), p < 0.05.*
4. *Measure of effect modification on the multiplicative scale and using students in the 12^th^ Grade as the reference group: 11^th^ Grade: 1.07 (0.88 – 1.26), p < 0.001; 10^th^ Grade: 1.11 (0.82 – 1.40), p < 0.001; 9^th^ Grade: 1.15 (0.74 – 1.55), p < 0.001.*

**Model 2: Does not include the mental health variable and substance usage**

**Complete data analysis**

**Modification of the effect of sexual minority status on bullying victimization by biological sex assigned at birth**

| **Total population analyses ^1^** | | | |
| --- | --- | --- | --- |
|  |  | **Percentage of being bullied** | **Adjusted OR (95% CI) ^2^** |
| **Heterosexual** | Male | 18.41% | 1.0, Reference |
|  | Female | 28.21% | 1.76 (1.64 - 1.89), *p* < 0.001 |
| **Sexual Minority** | Male | 35.15% | 2.44 (2.08 - 2.88), *p* < 0.001 |
|  | Female | 38.09% | 2.82 (2.51 - 3.18), *p* < 0.001 |
| **Stratified analyses by sex at birth ^1^** | | | |
|  | **Male** | Heterosexual | 1.0, Reference |
|  |  | Sexual Minority | 2.44 (2.08 - 2.87), *p* < 0.001 |
|  | **Female** | Heterosexual | 1.0, Reference |
|  |  | Sexual Minority | 1.62 (1.45 - 1.80), *p* < 0.001 |

***Note:***

1. *Total population analysis uses heterosexual males as the common reference group. Stratified analyses conducted separately for male and female students, both utilizing heterosexual students as their respective reference groups.*
2. *ORs were adjusted for race and grade.*
3. *Measure of effect modification on the additive scale (RERI): -0.38 (-0.81 - 0.05), p > 0.05*
4. *Measure of effect modification on the multiplicative scale: 0.66 (0.55 - 0.78), p < 0.001*

**Model 2: Does not include the mental health variable and substance usage**

**Complete data analysis**

**Effect of sexual minority status on bullying victimization by grade**

| **Total population analyses ^1^** | | | |
| --- | --- | --- | --- |
|  |  | **Percentage of being bullied** | **Adjusted OR (95% CI) ^2^** |
| **Heterosexual** | 12^th^ Grade | 19.9% | 1.0, Reference |
|  | 11^th^ Grade | 21.3% | 1.31 (1.21 – 1.40), *p <* 0.001 |
|  | 10^th^ Grade | 24.2% | 1.49 (1.36 – 1.66), *p* < 0.001 |
|  | 9^th^ Grade | 25.7% | 1.70 (1.47 – 1.97), *p* < 0.001 |
| **Sexual Minority** | 12^th^ Grade | 31.5% | 1.70 (1.32 – 2.20), *p* < 0.001 |
|  | 11^th^ Grade | 36.5% | 2.34 (2.00 – 2.73), *p* < 0.001 |
|  | 10^th^ Grade | 38.5% | 2.75 (2.37 – 3.19), *p* < 0.001 |
|  | 9^th^ Grade | 42.4% | 3.22 (2.69 – 3.86), *p* < 0.001 |
| **Stratified analyses by school grades ^1^** | | | |
|  | **12^th^ Grade** | Heterosexual | 1.0, Reference |
|  |  | Sexual Minority | 1.69 (1.38 – 2.06), *p* < 0.001 |
|  | **11^th^ Grade** | Heterosexual | 1.0, Reference |
|  |  | Sexual Minority | 1.84 (1.52 – 2.24), *p* < 0.001 |
|  | **10^th^ Grade** | Heterosexual | 1.0, Reference |
|  |  | Sexual Minority | 1.81 (1.46 – 2.24), *p* < 0.001 |
|  | **9^th^ Grade** | Heterosexual | 1.0, Reference |
|  |  | Sexual Minority | 1.88 (1.59 – 2.22), *p* < 0.001 |

***Note:***

1. *Total population analysis uses heterosexual students in 12^th^ Grade as the common reference group. Stratified analyses conducted separately for students in 12^th^, 11^th^, 10^th^, and 9^th^ Grade, both utilizing heterosexual students as their respective reference groups.*
2. *ORs were adjusted for race and sex.*
3. *Measure of effect modification on the additive scale (RERI) and using students in the 12^th^ Grade as the reference group: 11^th^ Grade: 0.34 (0.09 – 0.58), p < 0.01; 10^th^ Grade: 0.56 (0.10 – 1.01), p < 0.05; 9^th^ Grade: 0.82 (0.09 – 1/55), p < 0.05.*
4. *Measure of effect modification on the multiplicative scale and using students in the 12^th^ Grade as the reference group: 11^th^ Grade: 1.05 (0.88 – 1.26), p > 0.05; 10^th^ Grade: 1.08 (0.83 – 1.42), p > 0.05; 9^th^ Grade: 1.11 (0.78 – 1.59), p > 0.05.*

**Modification of the effect of sexual minority status on bullying victimization by biological sex assigned at birth, stratified by sexual identity group**

| **Sexual Minority \| Heterosexual ^1^** | | | |
| --- | --- | --- | --- |
|  |  | **Percentage of being bullied** | **Adjusted OR (95% CI) ^2^** |
| **Heterosexual** | Male | 18.54% | 1.0, Reference |
|  | Female | 27.98% | 1.46 (1.34 - 1.58), *p* < 0.001 |
| **Sexual Minority** | Male | 35.24% | 1.93 (1.63 - 2.24), *p* < 0.001 |
|  | Female | 38.71% | 1.78 (1.56 - 2.00), *p* < 0.001 |
| **Gay/lesbian \| Heterosexual** | | | |
|  |  | **Percentage of being bullied** | **Adjusted OR (95% CI) ^2^** |
| **Heterosexual** | Male | 18.41% | 1.0, Reference |
|  | Female | 28.16% | 1.49 (1.37-1.61), *p* < 0.001 |
| **Gay/Lesbian** | Male | 38.93% | 2.46 (1.73-3.19), *p* < 0.001 |
|  | Female | 33.66% | 1.49 (1.11-1.87), *p* < 0.001 |
| **Bisexual \| Heterosexual** | | | |
|  |  | **Percentage of being bullied** | **Adjusted OR (95% CI) ^2^** |
| **Heterosexual** | Male | 18.41% | 1.0, Reference |
|  | Female | 28.16% | 1.49 (1.36-1.61), *p* < 0.001 |
| **Bisexual** | Male | 35.20% | 1.74 (1.30-2.19), *p* < 0.001 |
|  | Female | 41.04% | 1.89 (1.61-2.16), *p* < 0.001 |
| **Not Sure \| Heterosexual** | | | |
|  |  | **Percentage of being bullied** | **Adjusted OR (95% CI) ^2^** |
| **Heterosexual** | Male | 18.41% | 1.0, Reference |
|  | Female | 28.16% | 1.49 (1.37-1.61), *p* < 0.001 |
| **Not Sure** | Male | 32.63% | 1.86 (1.41-2.31), *p* < 0.001 |
|  | Female | 33.96% | 1.65 (1.31-1.99), *p* < 0.001 |

***Note:***

1. *Total population analysis uses heterosexual males as the common reference group. Stratified analyses conducted separately for gay/lesbian students, bisexual students and students who answered not sure, all utilizing heterosexual students as their respective reference groups.*
2. *ORs were adjusted for race, grade, feeling sad or hopeless, and the use of the following products during the past 30 days: cigarettes, electronic cigarettes, and cigars.*
3. *Measure of effect modification on the additive scale (RERI): for* *sexual minorities: -0.62 (-0.96 - -0.28), p = 0.001; for gay/lesbian: -1.45 (-2.28 - -0.63), p = 0.001; for bisexual: -0.34 (-0.81-0.13), p= 0.15; for students answered “Not sure”: -0.7 (-1.27- -0.13), p = 0.016.*
4. *Measure of effect modification on the multiplicative scale: for sexual minorities: 0.63 (0.52 - 0.76), p < 0.001; for gay/lesbian: 0.41(0.27-0.61), p< 0.001; for bisexual: 0.73 (0.58-0.95), p= 0.02; for students answered “Not sure”: 0.59 (0.43-0.82), p< 0.001.*
5. *The proportion of subgroup in sexual minorities: heterosexual: 86.3%: gay/lesbian: 2.29%; bisexual: 7.51%; not sure: 3.92%.*

**Modification of the effect of sexual minority status on bullying victimization by grade, stratified by sexual identity group**

| **Sexual Minority \| Heterosexual ^1^** | | | |
| --- | --- | --- | --- |
|  |  | **Percentage of being bullied** | **Adjusted OR (95% CI) ^2^** |
| **Heterosexual** | 12^th^ Grade | 19.9% | 1.0, Reference |
|  | 11^th^ Grade | 21.3% | 1.43 (1.33 – 1.55), *p* < 0.001 |
|  | 10^th^ Grade | 24.2% | 1.73 (1.52 – 1.93), *p* < 0.001 |
|  | 9^th^ Grade | 25.7% | 2.07 (1.75 – 2.39), *p* < 0.001 |
| **Sexual Minority** | 12^th^ Grade | 31.5% | 1.32 (1.06 – 1.58), *p* = 0.007 |
|  | 11^th^ Grade | 36.5% | 1.99 (1.67 – 2.30), *p* < 0.001 |
|  | 10^th^ Grade | 38.5% | 2.43 (2.07 – 2.80), *p* < 0.001 |
|  | 9^th^ Grade | 42.4% | 2.98 (2.44 – 3.53), *p* < 0.001 |
| **Gay/lesbian \| Heterosexual** | | | |
|  |  | **Percentage of being bullied** | **Adjusted OR (95% CI) ^2^** |
| **Heterosexual** | 12^th^ Grade | 19.7% | 1.0, Reference |
|  | 11^th^ Grade | 21.3% | 1.43 (1.32-1.55), *p* < 0.001 |
|  | 10^th^ Grade | 24.4% | 1.72 (1.52-1.92), *p* < 0.001 |
|  | 9^th^ Grade | 25.6% | 2.06 (1.74-2.38), *p* < 0.001 |
| **Gay/Lesbian** | 12^th^ Grade | 31.9% | 1.17 (0.54-1.81), *p =* 0.61 |
|  | 11^th^ Grade | 25.9% | 2.06 (1.54-2.58), *p* < 0.001 |
|  | 10^th^ Grade | 40.3% | 2.73 (2.08-3.38), *p* < 0.001 |
|  | 9^th^ Grade | 44.1% | 3.61 (2.34-4.89), *p* < 0.001 |
| **Bisexual \| Heterosexual** | | | |
|  |  | **Percentage of being bullied** | **Adjusted OR (95% CI) ^2^** |
| **Heterosexual** | 12^th^ Grade | 19.7% | 1.0, Reference |
|  | 11^th^ Grade | 21.3% | 1.43 (1.32-1.55), *p* < 0.001 |
|  | 10^th^ Grade | 24.4% | 1.72 (1.51-1.92), *p* < 0.001 |
|  | 9^th^ Grade | 25.6% | 2.06 (1.73-2.38), *p* < 0.001 |
| **Bisexual** | 12^th^ Grade | 33.6% | 1.38 (0.95-1.80), *p =* 0.05 |
|  | 11^th^ Grade | 39.9% | 1.95 (1.59-2.31), *p* < 0.001 |
|  | 10^th^ Grade | 40.2% | 2.31 (1.89-2.74), *p* < 0.001 |
|  | 9^th^ Grade | 44.7% | 2.75 (2.11-3.39), *p* < 0.001 |
| **Not sure \| Heterosexual** | | | |
|  |  | **Percentage of being bullied** | **Adjusted OR (95% CI) ^2^** |
| **Heterosexual** | 12^th^ Grade | 19.7% | 1.0, Reference |
|  | 11^th^ Grade | 21.3% | 1.44 (1.32-1.55), *p* < 0.001 |
|  | 10^th^ Grade | 24.4% | 1.72 (1.52-1.92), *p* < 0.001 |
|  | 9^th^ Grade | 25.6% | 2.06 (1.74-2.38), *p* < 0.001 |
| **Not Sure** | 12^th^ Grade | 25.9% | 1.26 (0.76-1.76), *p =* 0.28 |
|  | 11^th^ Grade | 35.0% | 1.89 (1.51-2.28), *p* < 0.001 |
|  | 10^th^ Grade | 33.8% | 2.32 (1.89-2.75), *p* < 0.001 |
|  | 9^th^ Grade | 36.4% | 2.84 (2.14-3.55), *p* < 0.001 |

***Note:***

1. *Total population analysis uses heterosexual students in 12^th^ Grade as the common reference group. Stratified analyses conducted separately for gay/lesbian students, bisexual students and students who answered not sure, all utilizing heterosexual students as their respective reference groups.*
2. *ORs were adjusted for race, sex, feeling sad or hopeless, and the use of the following products during the past 30 days: cigarettes, electronic cigarettes, and cigars.*
3. *Measure of effect modification on the additive scale (RERI) and using students in the 12^th^ Grade as the reference group: for sexual minorities: 11^th^ Grade: 0.22 (0.03 – 0.42), p = 0.021; 10^th^ Grade: 0.39 (0.02 – 0.76), p = 0.041; 9^th^ Grade: 0.59 (-0.04 – 1.22), p = 0.067; for gay/lesbian: 11^th^ Grade: 0.45 (0.07-0.83), p = 0.021; 10^th^ Grade: 0.83 (0.0005,1.67), p = 0.05; 9^th^ Grade: 1.38 (-0.22 – 2.98), p = 0.09; for bisexual: 11^th^ Grade: 0.13 (-0.14-0.40), p =0.329; 10^th^ Grade: 0.22 (-0.28-0.72), p = 0.392; 9^th^ Grade: 0.31 (-0.51-1.13), p = 0.45; for students answered “Not sure”:11^th^ Grade: 0.20 (-0.08-0.48), p = 0.168; 10^th^ Grade: 0.34 (-0.21-0.89), p = 0.221; 9^th^ Grade: 0.52 (-0.42-1.46), p = 0.272.*
4. *Measure of effect modification on the multiplicative scale and using students in the 12^th^ Grade as the reference group: for sexual minorities: 11^th^ Grade: 1.04 (0.86 – 1.22), p < 0.001; 10^th^ Grade: 1.07 (0.79 – 1.35), p < 0.001; 9^th^ Grade: 1.09 (0.71 – 1.48), p < 0.001; for gay/lesbian: 11^th^ Grade:1.22 (0.76-1.69), p < 0.001; 10^th^ Grade: 1.35 (0.58-2.13), p = 0.001; 9^th^ Grade:1.50 (0.35-2.64), p = 0.011; for bisexual: 11^th^ Grade: 0.98 (0.77-1.20), p<0.001; 10^th^ Grade: 0.98 (0.65-1.30), p < 0.001; 9^th^ Grade: 0.97 (0.54-1.40), p < 0.001; for students answered “Not sure”: 11^th^ Grade:1.05 (0.77-1.32), p < 0.001; 10^th^ Grade:1.07 (0.65-1.50), p< 0.001; 9^th^ Grade: 1.10 (0.52-1.68), p < 0.001.*
5. *The proportion of subgroup in sexual minorities: heterosexual: 86.3%: gay/lesbian: 2.29%; bisexual: 7.51%; not sure: 3.92%.*
